# Assessing COVID-19 Booster Hesitancy and Its Correlates: An Early Evidence from India

**DOI:** 10.3390/vaccines10071048

**Published:** 2022-06-30

**Authors:** Geetanjali C. Achrekar, Kavita Batra, Yashashri Urankar, Ravi Batra, Naved Iqbal, Sabiha A. Choudhury, Deepti Hooda, Roohi Khan, Suraj Arora, Aditi Singh, Francesco Chirico, Manoj Sharma

**Affiliations:** 1 Department of Economics, GVMs College of Commerce and Economics, Ponda Goa 403401, India; geetanjali@gvmcommercecollege.ac.in; 2 Department of Medical Education, Kirk Kerkorian School of Medicine, University of Nevada, Las Vegas, NV 89102, USA; 3 Office of Research, Kirk Kerkorian School of Medicine, University of Nevada, Las Vegas, NV 89102, USA; 4 Community Health Centers of South-Central Texas, Gonzales, TX 78629, USA; urankary@chcsct.com; 5 Department of Information Technology, Coforge Ltd., Atlanta, GA 30338, USA; ravi.batra123@gmail.com; 6 Department of Psychology, Jamia Millia Islamia, New Delhi 110025, India; niqbal@jmi.ac.in; 7 Department of Psychology, Mizoram University, Tanhril, Aizaw l796004, India; sabihachoudhury9@gmail.com; 8 Department of Psychology, Maharshi Dayanand University, Rohtak 124001, Haryana, India; deepti.hooda@mdurohtak.ac.in; 9 Academics Department, London School of Commerce, Chaucer House, White Hart Yard, London SE1 1NX, UK; roohi5060@gmail.com; 10 Department of Restorative Dental Sciences, College of Dentistry, King Khalid University, Abha 61321, Saudi Arabia; surajarorasgrd@yahoo.co.in; 11 Department of Internal Medicine, Kirk Kerkorian, University of Nevada, Las Vegas, NV 89102, USA; aditi.singh@unlv.edu; 12 Post-graduate School of Occupational Health, Università Cattolica del Sacro Cuore, 00168 Rome, Italy; medlavchirico@gmail.com; 13 Department of Social and Behavioral Health, School of Public Health, University of Nevada, Las Vegas, NV 89119, USA; manoj.sharma@unlv.edu

**Keywords:** COVID-19, vaccine hesitancy, vaccine literacy, functional literacy, communicative literacy, critical literacy, vaccine confidence index, herd immunity, vaccine booster, SARS-CoV-2

## Abstract

The emergence of SARS-CoV-2 mutants, waning immunity, and breakthrough infections prompted the use of booster doses of the COVID-19 vaccine to fight against the pandemic. India started booster doses in January 2022 and it is critical to determine the intention of booster dose uptake and its correlates. Therefore, the current cross-sectional study aimed to investigate booster dose acceptability and associated predictors among the Indian population. A convenience sampling technique was utilized to recruit a sample of 687 Indian residents. A 55-item psychometric validated survey tool was used to assess booster dose acceptability, vaccine literacy and vaccine confidence. Univariate, bivariate, and multivariate statistical methods were used to analyze the data. Over 50% of participants reported their willingness to take the booster dose. Among the group not willing to take the booster dose (*n* = 303, 44.1%), a significantly larger proportion of respondents were unvaccinated with the primary series (12.2% vs. 5.2%, *p* < 0.001), had an annual income below 2.96 lacs/annum (52.8% vs. 33.1, *p* < 0.001), were residents of rural areas (38.0% vs. 23.2%, *p* < 0.001), were not living with vulnerable individuals (78.5% vs. 65.2%, *p* < 0.001) and did not have family/friends who had tested positive for COVID-19 (54.6% vs. 35.1%, *p* = 0.001). Demographic, vaccine variables and multi-theory model subscales to predict the initiation of booster dose among hesitant participants were statistically significant, R_2_ = 0.561, F (26, 244) = 11.978, *p* < 0.001; adjusted R_2_ = 0.514. Findings of this study highlight the need to develop evidence-based interventions to promote vaccine uptake, particularly among hard-to-reach communities living in developing countries.

## 1. Introduction

Most countries around the globe have considered the administration of additional booster doses of the COVID-19 vaccine given the emergence of new variants of SARS-CoV-2, concerns about waning immunity, and the incidence of breakthrough infections [1,2,3,4,5,6,7,8]. The side effects of the booster dose include systemic as well as local symptoms, including sore arm, headache, chills, tiredness, and nausea etc. [3,4]. These side effects are mostly mild and occur as a part of the immune response following the booster dose administration [3,4]. Reportedly, the effectiveness of the booster dose during the Omicron phase was 86% [9]. It was also found that the booster dose is 45% effective in preventing severe illness and hospitalizations following the Delta and Omicron infections [9]. Despite the scientifically proven and clinically tested vaccines across the world, vaccine hesitancy remains a big challenge in implementing vaccination drives by the government. The rates of vaccine hesitancy varied among countries, for instance, according to a recent United States (U.S.) based study, 42 percent of the Americans were not confident to take the booster dose of the COVID-19 vaccine [10]. The factors associated with booster dose hesitancy included but were not limited to lack of trust and demographic characteristics. Demographic factors, such as education status, marital status, regional differences, and political affiliation were among the main contributing factors of the booster dose hesitancy among the general population living in the U.S. [10]. A study related to vaccine acceptance among American healthcare workers (HCWs) found that only 7.9% of respondents were hesitant to take the primary series of the COVID-19 vaccine [11,12]. Individuals aged between 18 to 40 years, those with the lower educational attainment, and those lacking trust had higher vaccine hesitancy. Additionally, over 65 percent of the respondents had concerns about the vaccination’s effectiveness against new strains and the need to take booster doses [11,12].

A cross-sectional study among a Jordanian sample was undertaken to investigate how individuals having two doses of the primary series vaccination feel about being administered a booster dose and the factors associated with their decision [12]. Nearly 45 percent of the respondents were willing to take the booster dose and among the most frequently cited reasons by the participants were the presumption of low efficacy of the booster dose followed by the concerns surrounding short time interspersed between the administration of the primary series and booster dose [12]. Moreover, some participants, who acquired natural immunity subsequent to the prior COVID-19 infection did not feel the need to take a booster dose [12]. A cross-sectional study performed in Naples (Italy) investigated the mental readiness of a random sample of individuals, who had completed primary vaccination regime at immunisation sites to receive the booster of the COVID-19 vaccine. The booster dose acceptability was 85.7 percent in this Naples based sample [12]. Also, older adults who had a better health state following the primary vaccine series, those living with friends or someone among their family testing positive for COVID-19 and who had received the disease related information from official public institutions were willing to obtain the booster dose [12]. In a study performed among 31 Chinese provinces, a majority (93.7%) of the participants exhibited high acceptance in receiving the third dose of the vaccine. Individuals with lower levels of income, education, awareness and knowledge about COVID-19 were less likely to accept/take a third dose of COVID-19 [13]. This underscores the need to develop vaccine/health literacy campaigns and community outreach programs, especially within developing countries [14].

Scientists, researchers and policy planners view a booster dose as an effective way to fight the COVID-19 virus; however, public acceptance of a booster dose is relatively low due to a lack of trust and low vaccine confidence among people [15,16,17]. Vaccine misinformation prevailing on social media is an important factor contributing to vaccine hesitancy, which might have impacted the vaccine coverage rates and COVID response efforts at a global level [16,17]. This points to the importance of measuring vaccine initiation behaviours through tools grounded in the robust theoretical models, for instance, researchers in the U.S. used fourth generation theoretical frameworks (e.g., multi-theory model, MTM) to assess hesitancy towards primary series and booster doses among different demographic groups [18,19,20].

In India, the booster dose administration started in January 2022 [21] and there is a need to examine the booster dose acceptance levels and factors associated with hesitancy. Therefore, the objectives of the current study were twofold: first, to investigate the rate of booster dose acceptability and its correlates among the Indian population; second, to analyse the association between vaccine confidence, vaccine literacy and intention of initiating COVID-19 booster dose uptake based on the robust behavioural theory model.

## 2. Methods

### 2.1. Study Design, Participants, and Data Collection

This descriptive cross-sectional study utilized an online survey to recruit a sample of Indian nationals aged 18 years or above. Individuals who could understand the English language and were capable of providing informed consent were included. A convenience non-probability sampling was used to collect data during 13 December 2021–10 February 2022. The Qualtrics platform was used to deploy the survey and the survey link was shared with a wide variety of communities, social networks of the study investigators, social media, and other networking platforms, including WhatsApp, Facebook etc.

### 2.2. Ethical Considerations

This study was approved by the Institutional Human Ethics Committee of the Goa University (GU-DRDRM/IHEC-Cert/2021/03 dated 27 September 2021). Respondents’ consent was implied at the beginning of the study and potential respondents were informed that their participation in the study was voluntary and anonymous. Participants were given detailed information about the objectives, benefits and harms associated with this study, so they could make informed decisions about their participation. To abide by the ethical guidelines, no personal identifier information was collected to ensure anonymity.

### 2.3. Survey Instrument

A 55-item questionnaire consisting of psychometric valid tools was used in this study. The survey included 20 demographic and vaccine status related, 8 items to measure the Vaccine Confidence Index (VCI), 14 items to assess functional, communicative, and critical vaccine literacy [22,23], 13 items related to initiation of booster vaccination behavior based on the MTM [18,19,20]. The MTM tool has been used in a variety of other protective behaviors, such as mask wearing and hand-washing related to the COVID-19 [24,25]. The MTM tool includes subscales of “perceived advantages,” “perceived disadvantages,” “behavioral confidence,” and “changes in the physical environment.” [26]. All subscales were measured through 3 items each (12 items in total) [18,19,20]. One last item measured initiation, which was used as one of the dependent variables in this study. A 5-point Likert scale of frequency (ranging from “never” to “very often”) was used for the “perceived advantages” and “perceived disadvantages.” The summative scores of “perceived advantages” and “perceived disadvantages” were subtracted to calculate a derived subscale called “participatory dialogue.” The “Behavioral confidence” and “changes in physical environment” were measured on the 5-point Likert scale (“Not at all sure” to “Completely sure”) [18,19,20,26,27].

### 2.4. Sample Size Determination

Based on a 99% confidence level, a conservative 5% margin of error, and an estimated total adult population of 1 billion in India, 683 adults were required to make adequate inferences to the total population of adults in India. According to a previous study conducted in an Indian context [28], nearly 37% of the population was hesitant towards the COVID-19 vaccine, which we used as a proportion in a sample size calculation formula of *n* = (z) 2 *p* (1 − *p*)/d2 (d = margin of error; z = 1.96; and *p* = 0.37). The estimated sample size was 683 (621 + 10% non-response = 683) after accounting for 10% non-response.

### 2.5. Data Analysis

Data were cleaned and re-coded for the analysis. New variables to represent summative scores of vaccine confidence, vaccine literacy, and initiation were created. Data were assessed for distribution and outliers. Normally distributed continuous variables were represented as mean and standard deviation, whereas categorical variables were presented as counts and proportions. Given the independence of observations, chi-square and independent-samples-*t*-tests were used to compare categorical and continuous outcomes respectively among groups willing to take and not willing to take booster doses. A Pearson’s correlation analysis was conducted between main continuous variables, including age, vaccine literacy, vaccine confidence, and MTM constructs of initiation. A hierarchical linear regression was performed to investigate the predictive effect of vaccine literacy, confidence and MTM constructs on initiating the booster dose vaccination behavior after controlling the model for age, gender, marital status, education, income, region, if living with vulnerable individuals, and history of family/friends being COVID-19 positive. The polytomous variables were dummy coded before entering in the regression equation. Statistical significance for all tests and analyses was assumed a priori at *p* < 0.05. The Statistical Package for Social Sciences for Windows, version 27.0 (SPSS, Chicago, IL, USA) was utilized to analyze the data.

## 3. Results

**Figure 1:**
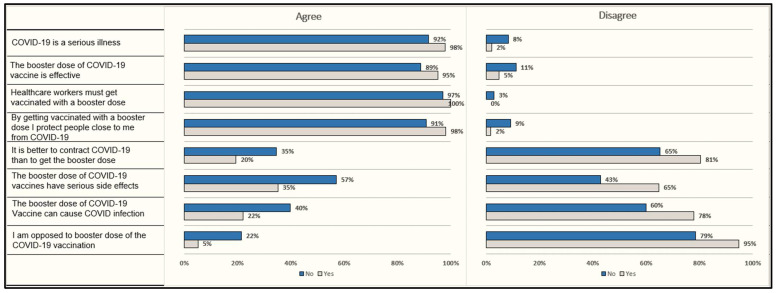
Item-wise comparison of vaccine confidence index among groups willing to take (yes) booster dose vs. those not willing to take (no) booster dose. Note: All differences were statistically significant at *p* < 0.05.

A total of 687 respondents provided consent and completed the survey. Over 90% of our sample reported being fully vaccinated and over 50% of the respondents indicated their willingness to take the booster dose (Table 1). Over 60% of the sample were females and never married. Participants were predominately from the north, north–west and Union territories of India. Approximately 50% of the sample reported having a bachelor’s degree and approximately 40% of the sample had an annual income below 2.96 lacs/annum (Table 1). Five of every ten participants reported having family/friends who had tested positive for COVID-19 and 1/4 of the participants had vulnerable individuals at home (Table 1). About 30% of the respondents were living in rural areas. Among the group not willing to take the booster dose (*n* = 303, 44.1%), a significantly larger proportion of respondents were unvaccinated with the primary series (12.2% vs. 5.2%, *p* < 0.001), had an annual income below 2.96 lacs/annum (52.8% vs. 33.1%, *p* < 0.001), were residents of rural areas (38.0% vs. 23.2%, *p* < 0.001), were not living with vulnerable individuals (78.5% vs. 65.2%, *p* < 0.001) and did not have family/friends who had tested positive for COVID-19 (54.6% vs. 35.1%, *p* = 0.001, Table 2). The mean scores of functional, communicative, critical, total vaccine literacies, and vaccine confidence were significantly higher among non-hesitant group as opposed to their hesitant counterparts (Table 3). Likewise, the mean score of “perceived advantages” of the booster dose vaccination was higher among the group willing to take it as compared to the group who was not willing to take the booster dose (8.56 ± 2.39 vs. 6.48 ± 2.46, *p* < 0.001). On the contrary, the booster hesitant group had higher mean scores of “perceived disadvantages” as compared to the non-hesitant ones (5.85 ± 2.34 vs. 4.75 ± 2.18, *p* < 0.001, Table 3). The mean scores of “behavioral confidence,” and “changes in the physical environment,” were also higher among the booster non-hesitant group as expected (Table 3). A significantly larger proportion of the non-hesitant group agreed that the booster dose is effective and protective against COVID-19 infections (Figure 1). On the other hand, booster hesitant respondents were opposed to the booster dose and think it may have serious side effects (Figure 1).

The Pearson correlation analysis indicated that “perceived advantages” are indirectly correlated with “perceived disadvantages” (r = −0.263, *p* < 0.001) and directly correlated with “behavioral confidence” (r = 0.547, *p* < 0.001), “changes in physical environment” (r = 0.506, *p* < 0.001), age (r = 0.123, *p* < 0.001), communicative vaccine literacy (r = 0.284 *p* < 0.001), critical literacy (r = 0.367, *p* < 0.001), and vaccine confidence (r = 0.462, *p* < 0.001). The “behavioral confidence” was strongly and directly correlated with the “changes in physical environment” (r = 0.792, *p* < 0.001), and moderately directly correlated with vaccine confidence (r = 0.447, *p* < 0.001). The “perceived disadvantages” were indirectly correlated with the vaccine confidence (r = −0.262, *p* < 0.01) (Table 4). As indicated in Table 5, the full model of demographic, vaccine variables and MTM subscales to predict initiation of the booster dose among hesitant participants (Model 5) was statistically significant, R^2^ = 0.561, F (26, 244) = 11.978, *p* < 0.001; adjusted R^2^ = 0.514. The addition of primary series vaccination status, vaccine literacy, and vaccine confidence to the prediction of booster dose acceptability (Model 2) led to a statistically significant increase in R^2^ of 0.095, F (3, 247) = 9.659, *p* < 0.001. The addition of “participatory dialogue” to the prediction of booster dose acceptability (Model 3) also led to a statistically significant increase in R^2^ of 0.153, F (1, 246) = 56.989, *p* < 0.001. The addition of “behavioral confidence” to the prediction of booster dose acceptability (Model 4) also led to a statistically significant increase in R^2^ of 0.130, F (1, 245) = 60.247, *p* < 0.001.

## 4. Discussion

The main objective of this study was to measure the factors associated with booster dose hesitancy in India, with an emphasis on vaccine literacy and vaccine confidence. The study was conducted after the approval of COVID-19 vaccine booster shots on 10th January 2022, by the Ministry of Health, Government of India. The emergence of coronavirus variants, concerns around waning immunity with primary vaccine series and evidence from other countries suggesting the benefits of additional doses to boost immunity were the primary drivers of initiating booster dose vaccination in India in January 2022 [29,30,31,32]. Over fifty percent (50%) of the participants in this study reported their willingness to take the booster dose against COVID-19, which is consistent with the findings of previous studies being conducted in other parts of the world [20,33,34]. Consistent with previous studies, our results indicate a strong association between socio-economic factors, demographic factors and vaccine acceptability [8,10,13,20,33,34]. With regards to gender, females were significantly more booster hesitant than males. The possible explanation may be attributed to persistent false messaging on social media about the association of COVID-19 vaccines with female infertility or birth defects [34,35,36].

Among other sociodemographic characteristics, participants with lower income, lower educational attainment, and those living in the rural areas were more hesitant to take the booster dose. Undoubtedly, rural–urban disparity has widened in COVID-19 vaccination status. Previous studies suggested that rural area residents have less general vaccine confidence and a few opportunities to gain access to health literacy regarding COVID-19 and vaccination [37,38]. Being culturally conservative and less educated, rural residents are more likely than urban residents to accept misinformation and conspiracy theories [38,39,40,41]. Additionally, the healthcare access in rural areas may not be adequate to provide large scale care to COVID-19 patients. This highlights the need to develop stronger healthcare infrastructure and culturally appropriate community education programs for the rural communities. This would need extensive collaboration between government and the members of the community, who may serve as trusted messengers or “COVID-19 vaccine ambassadors” [38,39]. To promote equitable access to vaccines, mobile vaccination units can be employed to target areas with lesser vaccination uptake in rural areas.

Next, the vaccine non-hesitant participants in our sample had significantly higher scores of functional, communicative, and critical literacies as opposed to hesitant participants. This finding is consistent with one study which was performed in Bangladesh, in which individuals with higher vaccine literacy had the greater vaccine acceptability [40]. This can be explained by the premise that individuals with high vaccine literacy will be able more efficient in distinguishing false from accurate information. The non-hesitant participants also had high mean scores of vaccine confidence and perceived advantages of booster doses. As self-reported by the non-hesitant participants, they perceived COVID-19 as a serious illness and also think that the booster dose of the COVID-19 vaccine is effective. These participants also believe that by taking booster dose, they can protect people close to them. This points to the sense of “collective responsibility” towards society and previous studies described it as a stronger predictor of vaccine initiation behavior [41].

Finally, all the MTM constructs of vaccine initiation behavior, i.e., participatory dialogue, behavioral confidence, and changes in physical environment were statistically significant predictors. This finding was consistent with a previous study which used MTM based assessment among college students, in which all the three MTM constructs were significant predictors of vaccine initiation behaviors [18]. These findings point to developing evidence-based interventions to initiate participatory dialogue between the “vaccine ambassador” or facilitators and community, emphasizing the advantages of vaccination over the disadvantages. To increase the behavioral confidence, “vaccine ambassadors “can use their own success stories as testimonials to help increase the vaccine uptake among hesitant individuals. Given a strong and positive correlation between behavioral confidence and vaccine confidence, the strategies outlined above will help in increasing vaccine confidence too. The construct of “change in the physical environment” can be intervened by increasing booster dose accessibility for the hard-to-reach communities, particularly those living in rural areas. In areas where the vaccine is freely accessible, reminder systems to ensure the completion of immunization through m-health intervention will be beneficial.

### Strengths and Limitations

To our best knowledge, this study is the first to investigate booster dose acceptability among the Indian population based on a robust theoretical model. However, this study is not without limitations. First, owing to the cross-sectional nature of the study, causal inferences were not made. Additionally, as the responses were digitally obtained, biases might have arisen from the housing conditions of the participants, in other words, environmental biases, were not controlled in this study. Next, the outcome might have been impacted by the social desirability bias. Moreover, due to differences in the characteristics of the eligible (e.g., ability to understand English and electronic access) and non-eligible participants, a “selection bias” might have been introduced into the study. The extent to which these factors would have influenced selection bias was difficult to quantify as we were unable to conduct a paper-based survey with a rural population lacking electronic access. At the time of data collection, India was hit hard by the third wave of the pandemic which limited our ability to conduct in-person surveys to minimize the effect of selection bias.

Next, the sample used in the study was not representative in terms of some demographic variables (e.g., age, marital status etc.), which might have impacted our ability to extrapolate the findings to the entire nation. Additionally, we expect to have “residual confounding” in this study as some variables, such as political and religion affiliation, were left unmeasured. Future studies can be planned to investigate the causal impact of the predictors being studied in the current investigation.

## 5. Conclusions and Implications

The universal acceptance of the COVID-19 booster dose among the diverse eligible populations across countries will facilitate the eradication of the pandemic in the near future. However, in reality booster dose vaccine hesitancy is still prevalent in many parts of the world due to various socio-economic and behavioral factors; governments across nations find this to be a challenge for wider vaccine coverage especially among the developing and poorer countries.

Despite the high acceptance levels among the sampled Indian population of booster dose uptake in this study, there is also a sizeable proportion of people who are vaccine hesitant. Being unvaccinated with the initial doses, having lower income levels, living in rural areas, not living with vulnerable individuals and not having family/friends who had tested positive were the strong predictors. This study provides new insights into the fact that high vaccine literacy and high vaccine confidence among the non-hesitant groups are strong predictors of booster dose vaccine acceptance levels.

There are important policy implications emerging from this study, which mainly highlight the need to create targeted educational campaigns to promote vaccine and health literacy among vaccine hesitant groups to dispel their myths and fears. This can be done by providing accurate and factual information about COVID-19 booster vaccination uptake to promote trust and acceptance in the vaccine. Additionally, among other countries with similar demographic profiles as India, it is critical to design effective communication strategies and active solutions to promote understanding about vaccine efficacy and effectiveness. For instance, vaccination uptake behavior promotion interventions can be developed, which will propagate information related to the effectiveness of the booster dose through community leaders and media. This will help increase the vaccine confidence among hesitant individuals, particularly in developing countries. Additionally, some studies have indicated that modest monetary incentives can be helpful in raising the vaccination rates [41].

## Figures and Tables

**Table 1:** Demographic characteristics of the respondents (*n*= 687).

Variable Name	Categories	*n* (%)	95% CI (LCL, UCL)
Vaccinated status (Primary Series)	Yes	630 (91.7)	89.4, 93.6
	No	57 (8.3)	6.3, 10.6
Will take COVID-19 booster dose	Yes	384 (55.9)	52.1, 59.6
	No/Not sure	303 (44.1)	40.4, 47.9
Age (Mean ± SD)	-	25.41 ± 9.1	24.7, 26.1
Gender	Male	224 (32.6)	29.1, 36.3
	Female	462 (67.2)	63.6, 70.7
Marital status	Married	116 (16.9)	14.2, 19.9
	Single, never married	433 (63.0)	59.3, 66.6
	Others	6 (0.8)	0.3, 1.9
Education	Bachelor’s degree	341 (49.6)	45.8, 53.4
	Master’s degree	197 (28.7)	25.3, 32.2
	Professional or trade school	28 (4.1)	2.7, 5.8
	Some high school or diploma	121 (17.6)	14.8, 20.6
Income	Below 2.96 lacs/annum	287 (41.8)	38.1, 45.5
	2.96 lacs–6.29 lacs/annum	197 (28.7)	25.3, 32.2
	6.29 lacs–29.6 lacs/annum	176 (25.6)	22.3, 29.1
	Above 29.6 lacs/annum	27 (3.9)	2.6, 5.7
Living with vulnerable individuals	Yes	189 (27.5)	24.2, 31.0
	No	460 (67.0)	63.3, 70.5
Family/friends tested COVID-19 positive	Yes	376 (54.7)	50.9, 58.5
	No	292 (42.5)	38.7, 46.3
Region	North	260 (37.8)	34.2, 41.5
	Northeast	25 (3.6)	2.4, 5.3
	South	23 (3.3)	2.1, 4.9
	West	235 (34.2)	30.6, 37.8
	Central	6 (0.9)	0.3, 1.9
	East	27 (3.9)	2.6, 5.7
	Union Territories	111 (16.2)	13.5, 19.1
Urbanity	Rural	204 (29.7)	26.3, 33.2
	Suburban	78 (11.4)	9.1, 13.9
	Urban	405 (59.0)	55.2, 62.6

Note: Some percentages may not add up to 100% as a few respondents preferred not to answer; SD = Standard deviation; CI = Confidence Interval; LCL = Lower Confidence Level; UCL = Upper Confidence Level.

**Table 2:** Bivariate comparison of Booster Dose acceptability by sample characteristics (*n* = 687).

Variable Name	Categories	Willing to Take BOOSTER		*p* Value	Statistics	ES
		Yes (*n* = 384, 55.9%)	No (*n* = 303, 44.1%)			
Vaccinated status (Primary Series)	Yes	364 (94.8)	266 (87.8)	**<0.001**	10.916	0.126
	No	20 (5.2)	37 (12.2)			
Age	-	26.45 ± 9.99	24.00 ± 7.77	**<0.001**	3.494	0.261
Gender	Male	136 (35.5)	88 (29.0)	0.073	3.216	0.068
	Female	247 (64.5)	215 (71.0)			
Marital status	Married	76 (19.8)	40 (13.2)	**0.009**	9.465	0.117
	Single, never married	244 (63.5)	189 (62.4)			
	Others	64 (16.7)	74 (24.4)			
Education	Bachelor’s degree	173 (45.1)	168 (55.4)	**0.022**	9.657	0.119
	Master’s degree	126 (32.8)	71 (23.4)			
	Professional or trade school	14 (3.6)	14 (4.6)			
	Some high school or diploma	71(18.5)	50 (16.5)			
Income	Below 2.96 lacs/annum	127 (33.1)	160 (52.8)	**<0.001**	33.705	0.221
	2.96 lacs–6.29 lacs/annum	114 (29.7)	83 (27.4)			
	6.29 lacs–29.6 lacs/annum	125 (32.6)	51 (16.8)			
	Above 29.6 lacs/annum	18 (4.7)	9 (3.0)			
Living with vulnerable individuals	Yes	130 (34.8)	59 (21.5)	**<0.001**	13.591	0.145
	No	244 (65.2)	216 (78.5)			
Family/friends tested COVID-19 positive	Yes	242 (64.9)	134 (45.4)	**<0.001**	25.341	0.195
	No	131 (35.1)	161 (54.6)			
Region	North	151 (39.3)	109 (36.0)	0.2	9.083	0.12
	Northeast	16 (4.2)	9 (3.0)			
	South	14 (3.6)	9 (3.0)			
	West	117 (30.5)	118 (38.9)			
	Central	5 (1.3)	1 (0.3)			
	East	19 (4.9)	8 (2.6)			
	Union Territories	62 (16.1)	49 (16.2)			
Urbanity	Rural	89 (23.2)	115 (38.0)	**<0.001**	19.049	0.167
	Suburban	43 (11.2)	35 (11.6)			
	Urban	252 (65.6)	153 (50.5)			

ES: Effect size; *p* values less than 0.05 are considered statistically significant and are bolded in the table.

**Table 3:** Comparing vaccine literacy, vaccine confidence, and MTM based initiation among groups with or without booster dose acceptability (*n* = 687).

Variable Name	Booster Dose Acceptability		*p* Value	Test Statistics	Effect Size
	Yes (*n* = 384)	No (*n* = 303)	-	-	-
Functional literacy	15.29 ± 3.19	13.87 ± 3.37	<0.001	5.679	0.43
Communicative literacy	16.02 ± 2.79	14.93 ± 2.99	<0.001	4.924	0.37
Critical literacy	12.85 ± 2.48	11.66 ± 2.75	<0.001	5.923	0.46
Total vaccine literacy	44.18 ± 5.74	40.46 ± 6.30	<0.001	8.070	0.62
Vaccine Confidence Index	2.53 ± 0.84	1.93 ± 0.79	<0.001	9.706	0.74
MTM based Initiation	3.05 ± 0.97	1.72 ± 1.03	<0.001	16.227	1.27
Perceived Advantages	8.56 ± 2.39	6.48 ± 2.46	<0.001	11.162	0.85
Perceived Disadvantages	4.75 ± 2.18	5.85 ± 2.34	<0.001	−6.352	0.48
Behavior Confidence	7.61 ± 2.87	3.85 ± 2.63	<0.001	17.683	1.35
Changes in the Physical Environment	7.91 ± 2.89	4.74 ± 2.73	<0.001	14.571	1.12

Note: All measures are represented as Mean ± standard deviation unless stated otherwise.

**Table 4:** Pearson correlations for study variables in the sample population (*n* = 687).

Variables	1	2	3	4	5	6	7	8	9
1. Advantages	1	−0.263 **	0.547 **	0.506 **	0.097 *	0.284 **	0.367 **	0.462 **	0.123 **
2. Disadvantages	0.263 **	1	−0.335 **	−0.288 **	−0.168 **	0.029	−0.053	−0.262 **	0.001
3. Behavioral Confidence	0.547 **	−0.335 **	1	0.792 **	0.178 **	0.240 **	0.313 **	0.447 **	0.140 **
4. Physical Environment	0.506 **	−0.288 **	0.792 **	1	0.235 **	0.270 **	0.344 **	0.454 **	0.144 **
5. Functional Literacy	0.097 *	−0.168 **	0.178 **	0.235 **	1	0.090 *	0.050	0.213 **	0.069
6.Iterative/Communicative Literacy	0.284 **	0.029	0.240 **	0.270 **	0.090 *	1	0.622 **	0.351 **	0.057
7. Critical Literacy	0.367 **	−0.053	0.313 **	0.344 **	0.050	0.622 **	1	0.292 **	0.073
8. Vaccine Confidence	0.462 **	−0.262 **	0.447 **	0.454 **	0.213 **	0.351 **	0.292 **	1	0.134 **
9. Age	0.123 **	0.001	0.140 **	0.144 **	0.069	0.057	0.073	0.134 **	1

** *p* < 0.01; * *p* < 0.05

**Table 5:** Hierarchical Multiple Regression (HRM) predicting intention of COVID-19 booster dose initiation among hesitant respondents (*n* = 303).

Variables	Model 1		Model 2		Model 3		Model 4		Model 5	
	B	*β*	B	*β*	B	*β*	B	*β*	B	*β*
Constant	1.240 *	-	−0.263	-	0.249	-	0.444	-	0.708	-
Age	0.022	0.153	0.018	0.128	0.014	0.099	0.009	0.063	0.002	0.014
Gender (Ref: Female)	−0.001	0.001	0.143	0.057	0.209	0.083	0.124	0.049	0.013	0.005
Education (Ref: Some high school or diploma)										
Bachelor’s degree	−0.081	−0.035	−0.182	−0.080	−0.143	−0.063	−0.127	−0.056	0.016	0.007
Master’s degree	−0.052	−0.020	−0.005	−0.002	0.143	0.054	0.071	0.027	0.174	0.066
Professional or trade school	−0.839 *	−0.158	−0.773 *	−0.145	−0.540	−0.102	−0.368	−0.069	−0.053	−0.010
Income (Ref: <2.96 Lacs/annum)										
2.96 lacs–6.29 lacs/annum	0.010	0.004	−0.079	−0.031	−0.152	−0.060	−0.047	−0.018	−0.061	−0.024
6.29 lacs–29.6 lacs/annum	0.069	0.024	−0.054	−0.019	−0.120	−0.041	−0.007	−0.002	−0.009	−0.003
Above 29.6 lacs/annum	−0.257	−0.036	−0.084	−0.012	−0.035	−0.005	−0.100	−0.014	−0.348	−0.049
Marital status (Ref: Married)										
Single	0.364	0.154	0.241	0.102	0.032	0.013	0.007	0.003	0.054	0.023
Others	0.334	0.124	0.199	0.074	0.042	0.016	0.111	0.041	0.170	0.063
Urbanity (Ref: Rural)										
Suburban	−0.542 *	−0.150	−0.544 *	−0.150	−0.140	−0.039	−0.161	−0.044	−0.168	−0.046
Urban	−0.143	−0.063	−0.129	−0.057	0.007	0.003	−0.119	−0.052	−0.169	−0.074
Living with vulnerable population (Ref: No)	−0.230	−0.083	−0.215	−0.078	−0.043	−0.015	0.033	0.012	0.003	0.001
Family/friends tested positive COVID−19	−0.016	−0.007	−0.076	−0.033	−0.064	−0.028	−0.019	−0.008	0.002	0.001
Region (Ref: West)										
Central	0.067	0.004	0.045	0.002	0.214	0.011	0.235	0.013	0.122	0.007
East	0.317	0.047	0.246	0.037	0.211	0.031	0.348	0.052	0.061	0.009
North	−0.137	−0.057	−0.169	−0.070	−0.020	−0.008	0.055	0.023	0.013	0.005
Northeast	−0.285	−0.045	−0.321	−0.051	−0.367	−0.058	−0.370	−0.058	−0.502	−0.079
South	0.780	0.116	0.559	0.083	0.542	0.081	0.663	0.099	0.623 *	0.093
Union Territories	−0.323	−0.106	−0.278	−0.091	−0.082	−0.027	−0.107	−0.035	−0.146	−0.048
Fully vaccinated with primary series (Ref: No)	-	-	0.479 *	0.143	0.339	0.101	0.151	0.045	−0.025	−0.007
Vaccine Confidence	-	-	0.324 **	0.231	0.074	0.053	0.003	0.002	−0.048	−0.034
Total Vaccine Literacy	-	-	0.018	0.098	0.013	0.070	0.006	0.035	−0.002	−0.011
Participatory dialogue	-	-	-	-	0.145 **	0.474	0.104 **	0.339	0.094 **	0.307
Behavioral confidence	-	-	-	-	-	-	0.179 **	0.417	0.070 *	0.164
Changes in the physical environment	-	-	-	-	-	-	-	-	0.181 **	0.441
R^2^	0.093	-	0.189	-	0.341	-	0.471	-	0.561	-
F	1.289	-	2.497 **	-	5.310 **	-	8.736 **	-	11.978 **	-
Δ R^2^	0.093	-	0.095	-	0.153	-	0.130	-	0.089	-
Δ F	1.289	-	9.659 **	-	56.989 **	-	60.247 **	-	49.657 **	-

* *p*-value < 0.05; ** *p*-value < 0.001; Adjusted R^2^ of initiation in the final model =0.514.

## Data Availability

The data presented in this study are available on request from the corresponding author. The data are not publicly available due to ethical reasons.
